# A Syndemic of Psychosocial Problems Places the MSM (Men Who Have Sex with Men) Population at Greater Risk of HIV Infection

**DOI:** 10.1371/journal.pone.0032312

**Published:** 2012-03-30

**Authors:** Wu Jie, Lu Ciyong, Deng Xueqing, Wang Hui, Hong Lingyao

**Affiliations:** Department of Medical Statistics and Epidemiology, School of Public Health, Sun Yat-sen University, Guangzhou, China; INSERM & Universite Pierre et Marie Curie, France

## Abstract

**Background:**

The MSM (Men who have sex with men) population suffers from very high rates of concurrent psychosocial problems. Together, these problems comprise a syndemic that increases the risk of HIV infection for this community. The precise mechanisms through which this syndemic can raise the likelihood of HIV infection warrant further exploration.

**Methodology/Principal Findings:**

A total of 522 MSM were enrolled via a multiframe sampling approach and were asked to report psychosocial problems, risky sexual behaviors and HIV test results. A count of psychosocial health problems was calculated to test the additive relationship of these factors on HIV risk. Adjusting analysis and restriction analysis were used to determine a proposed intermediate pathway. Psychosocial health problems are highly concurrent and intercorrelated among urban MSM. Greater numbers of health problems are significantly and positively associated with HIV infection, which is mediated, at least partially, by risky sexual behaviors.

**Conclusions/Significance:**

MSM experience concurrent psychosocial health problems that correlate with HIV infection in this community. We recommend the development of coping strategies for this population to deal with these psychosocial problems, both in prevention research and health policy.

## Introduction

Men who have sex with men (MSM) have been disproportionately affected by the human immunodeficiency virus (HIV) and continue to be major drivers of the HIV/AIDS epidemic throughout the world. Recent data indicate an emerging HIV epidemic among MSM in metropolitan areas of China [1]–[4]. By the end of 2007, it was estimated that 77,000 MSM were living with HIV/AIDS, accounting for 11% of the total number of estimated HIV cases in China [5]. MSM have recently emerged as a high-risk group in China.

As alarming as these epidemiological data are, even more disturbing is the lack of preparedness to address HIV risk and prevention in this population. Modifying the health behaviors of individuals at risk is a complex challenge. Not only are there interpersonal challenges and life experiences to overcome, but there are also social, psychological and cultural obstacles to surmount in curtailing the epidemic.

In certain areas, homosexuality has traditionally been stigmatized. Therefore, MSM in these locations are vulnerable to a variety of psychosocial difficulties [6], and researchers have suggested that homophobia and prejudice contribute to these psychosocial difficulties through a process termed minority stress. This model incorporates various psychological and stress theories to explain the health disparities experienced by MSM and other sexual minorities [7]. External social conditions and structures, such as stigma and discrimination toward same-sex behavior, serve as distal stressors that impact an individual through more proximal processes, including expectations of rejection, concealment and internalized homophobia. In turn, these individuals may subsequently present with depression, anxiety and suicidal ideation [7], [8]. These psychosocial health problems then interact with HIV risk to produce a syndemic [9].

According to the Centers for Disease Control and Prevention (CDC), a syndemic is defined as two or more epidemics (i.e., notable increases in the rate of specific diseases in a population) interacting synergistically and contributing, as a result of their interaction, to an excess burden of disease in a population [10]. Medical anthropologist Merrill Singer recognized the first syndemic, the Substance Abuse, Violence and AIDS (SAVA) syndemic, among inner-city residents of Hartford. He found that a collection of tightly interwoven circumstances, including poverty, loss of adequate housing, family instability, drug-related violence and inequitable health care, fueled the increased risk of AIDS among this population [11], [12]. Stall subsequently identified the syndemic model for polysubstance abuse, depression, childhood sexual abuse and partner violence in MSM [9].

This model provides a framework for understanding these synergistic relationships by identifying these factors as mutually interacting, influential issues that contribute to HIV infection in MSM. However, little research has been conducted to describe this syndemic in Chinese MSM.

Our primary objective was to determine whether this particular syndemic was associated with HIV infection in Chinese MSM and to determine which psychosocial problem was most closely related to HIV infection. For example, because individuals who experience intimate partner violence and childhood sexual abuse often develop posttraumatic stress disorder (PTSD), PTSD was significantly associated with having engaged in unprotected anal sex and acquiring HIV [13]–[15]. In this study, we divided 5 psychosocial health problems into 3 categories: substance abuse (polysubstance abuse and binge drinking), depression and risk factors for PTSD (partner violence and childhood sexual abuse). Our goal was to determine which psychosocial problems have the strongest relationship with HIV infection. We also aimed to assess whether psychosocial problems tend to have an intermediate effect or an independent cofactor effect on high-risk sexual behavior.

## Methods

### Participants and procedures

One of the greatest methodological challenges in the scientific study of gay and bisexual individuals is obtaining a representative sample. We used a multiframe sampling approach to avoid bias resulting from reliance on a single sampling source. Trained outreach workers from the MSM community randomly approached potential study participants in target venues (e.g., gay bars and public parks) and invited them to participate in this study. Advertisements were posted on the website for the Lingnan Partner Support Center (LPSC), which has over 10,000 registered users and 1,000 visits per day. Word-of-mouth referrals by participants were also used to recruit study subjects. Participants were screened via telephone by trained study staff and were deemed eligible if they were male, were 18 years of age or older and reported having sex with men. Participants were compensated $20 for their participation in the study.

The study was performed between March and August of 2010. A total of 522 participants completed a self-administered questionnaire and gave a blood sample of 3–5 ml for HIV testing. All study activities took place at the Lingnan Partner Support Center (LPSC), a freestanding health care and research facility specializing in HIV/AIDS care and serving the needs of the MSM community in Guangzhou, China.

The nature and purpose of the study was explained to each participant after they provided written consent to participate. The survey was anonymous; no names or other personal identifying information were collected from the participants. Each participant was given a unique study number that was used to connect the HIV test results to the subject. A private room at the LPSC was used for the interviews, which took approximately 30 minutes.

### Ethics

The Sun Yat-sen University Institutional Review Board approved the study, and each study participant provided written informed consent with a trained researcher.

### Measures

#### Demographic and background characteristics

Participants were asked to report their age, ethnicity, educational level, employment status, marital status and sexual orientation. All other variables used in the analysis are described in Table 1.

**Table 1:** Demographic characteristics.

Characteristics	N	%
**Age (years), median (range)**	28(18–68)	
**Ethnicity**		
Han	507	97.1
others	15	2.9
**Household registration**		
local	248	47.7
other	274	52.3
**SES**		
low	87	16.7
middle	309	59.2
high	126	24.1
**Education**		
At least junior college	399	76.4
Less than junior college	123	23.6
**Sexual orientation**		
gay	378	72.4
bisexual	96	18.4
uncertain	48	9.2
**employment**		
Full/part time	448	85.8
unemployed	74	14.2
**Marital status**		
Married	68	13.0
Unmarried	454	87.0
**HIV**		
Positive	18	3.4
Negative	504	96.6

SES, socioeconomic status.

#### Drug use

We defined drug use as the use of one or more recreational drugs (e.g., marijuana, cocaine, heroin, hallucinogens, amphetamines, methamphetamine, MDMA [“ecstasy”], or ketamine) in the previous 6 months.

#### Binge drinking

Binge drinking has been defined by the National Institute on Alcohol Abuse and Alcoholism (NIAAA) and other studies as the consumption of five or more drinks per occasion or sitting for men. We assessed binge drinking by asking participants, “How often during the past 6 months did you have 5 or more drinks per day?” Those who reported consuming 5 or more drinks at least one day per week were considered to have engaged in binge drinking (coded 1; those not reporting binge drinking were coded 0).

#### Depression

The Center for Epidemiological Studies–Depression (CES-D) scale was utilized to assess for depression, and scores greater than 22 indicated depression. Respondents used 4-point scales to indicate the number of days in the previous week that each depressive feeling occurred (Cronbach's alpha, 0.97).

#### Childhood sexual abuse

Childhood sexual abuse was defined as the experience of being “forced or frightened by someone into doing something sexually” before age 16 by someone at least 5 years older than the participant.

#### Intimate partner violence

Intimate partner violence (IPV) was assessed with three items asking about experiences of being threatened, physically hurt, or bullied by a current or former same-sex partner. People who reported any of these items were considered to have experienced IPV.

#### Syndemic

The number of psychosocial risk factors was calculated by summing the scores for each of the five dichotomous psychosocial health problems: drug use, binge drinking, depression, intimate partner violence and childhood sexual abuse. We have named this count of psychosocial risk factors the syndemic variable.

#### Sexual behavior

Men reported their sexual behavior during the preceding 6 months. A man was considered to have multiple partners if he had more than one anal sex partner. The men were asked about condom usage using a Likert scale with options ranging from 1 (never) to 4 (always). Men who responded with any number other than 4 were considered to have participated in unprotected sex.

### Statistical analysis

The primary goal of this analysis was to test whether the presence of certain psychosocial factors results in syndemically increased vulnerability to HIV infection in Chinese MSM. Bivariate odds ratios were computed for each of the psychosocial factors, sexual risk behaviors and HIV outcomes. Logistic regression was employed to test for the relationship between psychosocial factors and HIV infection. The results are reported as odds ratios (ORs) with 95% confidence intervals (CIs).

To determine the relative importance of each of the major components of the syndemic variable on HIV infection, we categorized the psychosocial variables into substance use (binge drinking and drug use), depression and PTSD risk factors (IPV and childhood sexual abuse).

If a psychosocial variable was associated with HIV infection, we conducted additional analyses to evaluate the mechanism of the relationship; that is, we sought to determine whether the relationship was mediated by sexual risk behaviors, a cofactor effect or both. Typically, epidemiologists test a mediation hypothesis by adjusting the risk factor effect estimate for the proposed mediator(s); a change in the OR towards null (i.e., towards one) provides evidence for the proposed intermediate pathway [16].

In addition, we performed a subanalysis using restriction. For infectious diseases, causal evidence for a cofactor effect is best obtained with one condition of exposure to infection [17]. Therefore, this subanalysis was restricted to the 432 men who reported engaging in unprotected sex and having multiple anal sex partners.

## Results

A total of 530 MSM were recruited for the study, and 522 (95.4%) completed the questionnaire and provided blood samples. Eight refused to finish the questionnaire but consented to give blood for the HIV test. The most common reason for refusing the questionnaire was that the participant was too busy to complete it. Table 1 describes the demographic characteristics and HIV infection status of this sample. The age of the participants ranged from 18 to 68 years, with a median age of 28 years. More than half (55%) of the participants were between 26 and 35 years of age. The majority were educated beyond junior college (76.4%) and were employed. Eighty-four percent of participants were unmarried. Most participants identified themselves as gay (72.4%), and a minority identified as bisexual (18.4%). The remainder were uncertain about their sexual orientation (9.2%). The overall prevalence of HIV in this sample was 3.4%.

Table 2 describes the rates of psychosocial variables, sexual risk behaviors, and HIV for the sample. Approximately 35% and 3.8% of the sample reported binge drinking and using drugs in the previous 6 months, respectively. In addition, 3.4% of the sample tested positive for HIV, and over half of the sample reported engaging in each HIV risk behavior. The extent to which the psychosocial factors are related to each other is notable; for example, drinking was associated with drug abuse and multiple anal sex partners. Men who had certain psychosocial factors were more likely to report risky sexual behaviors. For instance, participants who reported depression had over two times the odds of having had multiple anal sex partners.

**Table 2:** Logistic regression model to evaluated the association between psychosocial health problems.

	1.Binge drinking	2.Drug abuse	3.depression	4.Childhood sex abuse	5.Partner violence	6.Multiple Anal Sex Partners	7.Unprotected Anal Sex	8.HIV positive
Prevalence	35.6%	3.8%	33%	15.1%	2.7%	54.3%	62.8%	3.4%
2	2.8(1.1–7.1)							
3	-	-						
4	-	-	1.6(1.1–2.3)					
5	-	-	3.8(1.3–11.6)	4.5(1.5–13.3)	-			
6	1.5(1.0–2.2)	5.0(1.5–17.3)	-	-	-			
7	-	-	2.0(1.0–4.0)	2.1(1.2–3.6)	4.4(1.4–14.2)	-		
8	-	-	2.6(1.0–6.8)	3.8(1.4–10.2)	-	2.7(1.1–7.1)	4.9(1.1–21.7)	

Note. Dash indicates odd ratio is nonsignificant. All odds ratios shown, two-tailed *p*<.05.

Our next set of analyses focused on the relationship between the syndemic variable and vulnerability to HIV infection, controlling for demographic variables such as age and socioeconomic status (SES) (see Table 3). The unadjusted regression model shows that the presence of these psychosocial variables was associated with participation in high-risk sex (OR = 1.8; 95% CI = 1.1–3.0).

**Table 3:** Logistic regression models to evaluate the association between syndemic and HIV infection.

items	OR unadjusted (95% CI)	OR adjusted (95% CI)^a^	OR restricted (95% CI)^b^
Age	1.0(0.9–1.1)	1.0(0.9–1.1)	1.0(0.9–1.1)
SES			
low	1.0	1.0	1.0
middle	0.5(0.1–1.6)	0.4(0.1–1.5)	0.6(0.2–2.5)
high	0.7(0.2–2.8)	0.7(0.2–2.7)	0.9(0.2–4.2)
Syndemic#	1.8(1.1–3.0)	1.5(1.0–2.9)	1.4(0.9–2.8)

Unconditional logistic regression. CI, confidence interval; OR, odds ratio. SES, socioeconomic status.

a Adjusted for sexual risk behaviours; having sex with multiple partners, anal sex without a condom or with partial condom use.

b Restricted to 432 men who engaged in the highest risk behaviours; having sex with multiple partners, anal sex without a condom or with partial condom use.

# OR refers to each unit increase of the syndemic score confers approximately corresponding increase in odds of HIV infection compared to the previous syndemic score.

When sexual behavioral risk factors were added to the unconditional logistic regression models, the magnitudes of the ORs decreased towards the null (Table 3, second column). The syndemic variable remained significantly associated with the risk of HIV infection in the adjusted model, but the magnitude of the OR was decreased (OR = 1.5; 95% CI = 1.0–2.9). In the restriction analysis, the syndemic effect size was further attenuated and became statistically insignificant (OR = 1.4; 95% CI = 0.9–2.8).

To determine the relative importance of the major components of the syndemic variable, a multivariate logistic regression analysis was conducted with HIV as the dependent variable and substance use (binge drinking and drug use), depression and PTSD risk factors (partner violence and childhood sexual abuse) as the independent variables. Table 4 shows that in the unadjusted analysis, PTSD symptoms significantly increased the odds of testing positive for HIV (OR = 3.5, *P*<0.05), and depression also increased the risk of HIV (OR = 2.9, *P*<0.05). The relationship between substance abuse and HIV was reversed (OR = 0.8), but the *P* value was above 0.05. The OR of PTSD risk factors in the adjusted and restricted analysis decreased. The OR of depression decreased in the adjusted analysis but increased in the restricted analysis (see Table 4).

**Table 4:** Logistic regression models to evaluate the association between syndemic components and HIV infection.

Types of psychosocial problems	OR unadjusted (95% CI)	OR adjusted (95% CI)^a^	OR restricted (95% CI)^b^
Substance abuse	0.8(0.3–2.3)	0.8(0.3–2.3)	0.9(0.3–2.4)
depression	2.9(1.1–7.6)	2.9(1.1–7.6)	3.3(1.2–4.3)
trauma	3.5(1.3–9.4)	3.1(1.1–8.6)	2.8(0.9–8.0)

Unconditional logistic regression. CI, confidence interval; OR, odds ratio. SES, socioeconomic status.

a Adjusted for sexual risk behaviours; having sex with multiple partners, anal sex without a condom or with partial condom use.

b Restricted to 432 men who engaged in the highest risk behaviours; having sex with multiple partners, anal sex without a condom or with partial condom use.

## Discussion

Our study supports the previous evidence that additive psychosocial health problems—collectively known as a syndemic—exist among urban MSM. In our study the interconnection of these psychosocial problems magnified the effects of the HIV/AIDS epidemic in this population. In addition, we found that this effect is at least partially mediated by high-risk sexual behavior. We found that not only is sexual risk behavior an important mechanism underlying the relationship between the syndemic of psychosocial problems and HIV infection for gay and bisexual men, but it also partially mediates the syndemic effects of psychosocial problems on HIV infection.

Given the cross-sectional nature of the study design, some results should be interpreted with caution; for instance, depression and substance-use may be the result of HIV-infection in some men rather than a cause. All relationships identified in this study are restricted to bidirectional relationships, not causality.

In the mediation analysis, the size of the syndemic effect was not attenuated to one after adjusting for sexual risk behaviors, which may be explained by incomplete adjustment and residual confounding by unmeasured sexual behaviors. Another possible explanation is that the syndemic of psychosocial problems acts as a cofactor independent of its effect on sex behaviors. If any cofactor effect exists, it is not strong, given the weak and statistically insignificant associations we observed once the effect of sexual behavior was removed by restriction analysis.

If cofactor effects indeed exist, this may be due to differences in sexual networks. MSM have few places to meet with each other. Gay bars are one of the most visible and accessible places to meet, but the omnipresent alcohol and sexually charged environment may contribute to substance use and risky sexual behaviors [18]. The biological effects of psychosocial problems on susceptibility to illness may also explain any cofactor effects. Research conducted among people who are already infected has revealed the biological effects of psychosocial problems on HIV infection. Depression, stressful life events and trauma are associated with a decrease in CD4 T-lymphocytes, an increase in viral load, faster clinical decline, and higher AIDS-related and all-cause mortality [19]. To the best of our knowledge, few studies, if any, have been conducted on the effect of psychosocial problems on biological markers of HIV susceptibility in high-risk populations.

Mediation analysis requires careful consideration of potential confounders of the relationships between a potential risk factor and the proposed mediator and between the mediator and the outcome [20], [21]. We adjusted for all demonstrated sexual risk factors in this population as well as other sexual risk behaviors that confer a lower risk, in accordance with the epidemiological literature. We also used restriction analysis to provide corroborative evidence for the results derived from the adjustment approach. We performed an analysis restricted to men who engaged in the highest risk behavior, thereby creating a condition for (possible) exposure to HIV [17]. We believe that the results of this restriction analysis provide the strongest evidence for the mediation hypothesis.

Multivariate analyses suggest that depression and the experience of trauma show the strongest relationship to HIV infection a finding that is partially mediated by risky sexual behaviors. Our results are partially consistent with those from Mustanski's study [22], which showed that violence (known as PTSD risk factors in our study) has the strongest relationship with HIV. However, substance abuse was not significantly associated with HIV in our study in contrast to Mustanski's study. This difference may be explained by the fact that substance use is rare among MSM in China.

This is the first study to investigate the mediating role of sexual risk behaviors on the syndemic relationship between psychosocial problems and HIV infection. The finding that the MSM population experiences multiple, concurrent psychosocial problems that interact to form a syndemic helps to explain why many patients in STD clinics find it difficult to change their sexual behaviors. It may be difficult for people to focus on sexual behavior changes when they are faced with numerous other daily problems and competing life priorities. Health care providers should be prepared to address MSM who experience a range of mental health, substance use and behavioral problems.

The data presented here should be interpreted with the following limitations in mind. First, the cross-sectional design cannot identify the temporal order between exposure and outcomes, which limits our ability to make causal inferences about the relationship between independent variables and HIV infection. Therefore, in this study we can only identify an association and not causality. Additionally, our sample is not a random sample, although it is believed to be representative of the local population of MSM. Generalizing the results to a more diverse MSM population should be conducted with caution. Third, because the duration of HIV infection of the men in our study is not known, we do not know if their sexual behavior in the last six months is relevant to their HIV infection. Finally, recall bias and social desirability bias are possible because the information on drug use and sexual behavior was collected by self-report. The majority of the information on sexual behaviors in the last 6 months was used in the data collection.

In sum, intimate partner violence, childhood sexual abuse, alcohol use, drug use, depression and high-risk sexual behavior frequently coexist among gay and bisexual men, and experiencing more of these psychosocial health problems was associated with greater odds of being infected with HIV. Thus, our data support the development of new sexual risk reduction interventions and the refinement of existing ones that MSM can incorporate using multi-faceted approaches, including mental health treatment and psychosocial intervention. Additional studies, such as case-control studies and cohort studies, should be performed to demonstrate the importance of syndemics in the acquisition of HIV among the MSM population.
